# Susceptibility of HIV-1 Subtypes B′, CRF07_BC and CRF01_AE that Are Predominantly Circulating in China to HIV-1 Entry Inhibitors

**DOI:** 10.1371/journal.pone.0017605

**Published:** 2011-03-11

**Authors:** Xiaoling Yu, Lin Yuan, Yang Huang, Weisi Xu, Zhiming Fang, Shuwen Liu, Yiming Shao, Shibo Jiang, Liying Ma

**Affiliations:** 1 State Key Laboratory for Infection Disease Prevention and Control, National Center for AIDS/STD Control and Prevention (NCAIDS), Chinese Center for Disease Control and Prevention (China-CDC), Beijing, China; 2 School of Pharmaceutical Sciences, Southern Medical University, Guangzhou, China; 3 Lindsley F. Kimball Research Institute, New York Blood Center, New York, New York, United States of America; 4 Key Laboratory of Medical Molecular Virology of MOE/MOH and Institute of Biomedical Sciences, Shanghai Medical College, Fudan University, Shanghai, China; University of Minnesota, United States of America

## Abstract

**Background:**

The B′, CRF07_BC and CRF01_AE are the predominant HIV-1 subtypes in China. It is essential to determine their baseline susceptibility to HIV entry inhibitors before these drugs are used in China.

**Methodology/Principal Findings:**

The baseline susceptibility of 14 representative HIV-1 isolates (5 CRF07_BC, 4 CRF01_AE, and 5 B′), most of which were R5 viruses, obtained from drug-naïve patients to HIV entry inhibitors, including two fusion inhibitors (enfuvirtide and C34), two CCR5 antagonists (maraviroc and TAK779) and one CXCR4 antagonist (AMD3100), were determined by virus inhibition assay. The sequences of their *env* genes were amplified and analyzed. These isolates possessed similar susceptibility to C34, but they exhibited different sensitivity to enfuvirtide, maraviroc or TAK779. CRF07_BC isolates, which carried polymorphisms of A578T and V583I in the N-terminal heptad repeat and E630Q, E662A, K665S, A667K and S668N in the C-terminal heptad repeat of gp41, were about 5-fold less sensitive than B′ and CRF01_AE isolates to enfuvirtide. Subtype B′ isolates with a unique polymorphism site of F317W in V3 loop, were about 4- to 5-fold more sensitive than CRF07_BC and CRF01_AE isolates to maraviroc and TAK779. AMD3100 at the concentration as high as 5 µM exhibited no significant inhibitory activity against any of the isolates tested.

**Conclusion:**

Our results suggest that there are significant differences in baseline susceptibility to HIV entry inhibitors among the predominant HIV-1 subtypes in China and the differences may partly result from the naturally occurring polymorphisms in these subtypes. This study provides useful information for rational design of optimal therapeutic regimens for HIV-1-infected patients in China.

## Introduction

The human immunodeficiency virus type 1 (HIV-1) can be classified to three major groups, M (major), O (outlier) and N (non-M non-O or new). The M group, which has caused the vast majority of HIV-1 infections worldwide, can be further divided into several subtypes, including A–D, F–H, J and K, as well as several circulating and unique recombinant forms (CRFs and URFs) [1], [2]. The greatest genetic diversity of HIV-1 subtypes has been found in China. Among them, HIV-1 subtype B′ (also known as Thai B), CRF07_BC (BC) and CRF01_AE (AE) are the predominant circulating viruses in China [3], [4].

HIV-1 infection is established after viral entry into the target cell [5]. The molecules involved in HIV-1 entry are attractive targets for developing antiviral therapeutics [6]–[8]. Based on drug targets, the HIV-1 entry inhibitors can be classified into three groups, including i) attachment inhibitors (e.g., NBD556 and BMS378806) that block the interaction between the HIV-1 envelope glycoprotein (Env) surface subunit gp120 and CD4 receptor by targeting to the CD4-binding site on gp120; ii) co-receptor antagonists, which block the interaction ligand between gp120 and CCR5 (e.g., UK-427857 and TAK779) or CXCR4 (e.g., AMD3100); and iii) HIV-1 fusion inhibitors (such as T20 and C34) [9], [10]. T20 (brand name: Fuzeon; generic name: enfuvirtide) and UK-427857 (brand name: Selzentry; generic name: maraviroc) were approved by the US FDA in 2003 and 2007 as the first and second HIV-1 entry inhibitors, respectively, for treatment of HIV-1-infected patients who fail to respond to the current antiretroviral drugs (ARVs) [11], [12].

The Chinese national AIDS treatment program, including the free treatment with nucleotide and nucleoside reverse transcriptase inhibitors (NRTIs), non-nucleoside reverse transcriptase inhibitors (NNRTIs) and protease inhibitors, has significantly reduced the mortality rate among HIV-1 infected patients [13], [14]. However, the continuous emergence of HIV-1 resistance to NRTIs and NNRTIs has resulted in high failure rate in clinical applications of these anti-HIV drugs [15]–[17]. In order to improve the outcome of the treatment and to prevent the transmission of resistant strains, it is urgently needed to design new effective treatment regimens for those who have failed to respond to the first line ARVs. HIV entry inhibitors could be the first choice for these patients in China. However, it is unclear whether these HIV entry inhibitors are also highly effective against the predominant HIV-1 strains circulating in China since none of the US FDA-approved HIV entry inhibitors has ever been tested in clinics in China.

The present study aims to test the baseline susceptibility of the predominant HIV-1 subtypes circulating in China to HIV entry inhibitors and characterize the genotype polymorphisms in these subtypes. This study is expected to provide a clearer understanding of the natural resistance of the predominant viruses to HIV entry inhibitors and valuable information for rational design of treatment regiments containing HIV entry inhibitors for HIV-infected patients in China and other Asian countries.

## Results

### Characteristics of the study population and HIV-1 variants

**Figure 1:**
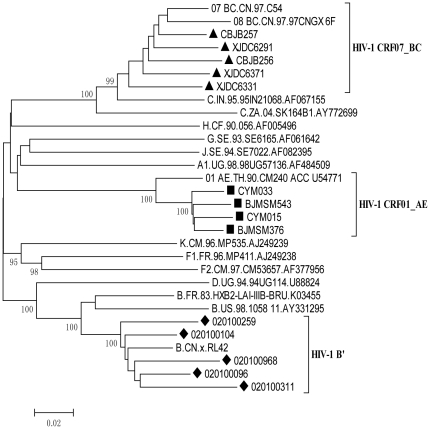
Phylogenetic analysis of HIV-1 *env* genes. The viral *env* sequences were obtained by PCR analysis of plasma samples of 14 HIV-1 infected and treatment naïve patients. “▪”, “▴”, “⧫” represent the *env* sequence of CRF07_BC, CRF01_AE and B′ strains tested respectively.

We isolated 26 viral strains with *in vitro* infectivity from peripheral blood mononuclear cells (PBMCs) of the HIV-1-infected patients. But in this study, we only used in this study 14 strains isolated from the patients who had not used ARVs before, including 11 males and 3 females (averaging 37.6 years old). They were infected by HIV-1 through three different pathways, including former plasma donors (FPD) from Anhui Province (n = 5), injection drug users (IDUs) from Xinjiang province (n = 5) and the victims of sexually transmitted infections (STIs) from Beijing (n = 4). As shown in Table 1, the average viral load was 5.14±0.97 log copies/mL (ranged from 4 to 6.2 log copies/mL), and the average CD4 count was 415±185 cells/mL (ranged from 75 to 628 cells/mL). The infection strains belong to different HIV-1 subtypes, including 5 B′, 5 CRF07_BC and 4 CRF01_AE isolates (Table 1). All of the isolated strains used CCR5 as co-receptor, including 2 dual tropic (R5/X4-tropic) isolates. The phylogenetic tree was then constructed (Fig. 1).

**Table 1:** Characteristics of the HIV-1 clinical isolates that are predominantly circulating in China.

Virus	Sex	Age	CD4 counts (/µl)	CD8 counts (/µl)	Viral load (log_10_)	Coreceptor	Subtype
CBJB257	F	27	376	1,122	4.0	R5	CRF 07_BC
XJDC6291	M	35	155	1,596	6.1	R5	CRF 07_BC
CBJB256	M	46	75	352	6.0	R5	CRF 07_BC
XJDC6371	M	34	590	1,955	5.5	R5	CRF 07_BC
XJDC6331	M	38	319	1,435	6.2	R5	CRF 07_BC
CYM033	M	28	397	918	3.0	R5	CRF 01_AE
BJMSM543	M	28	613	766	5.8	R5	CRF 01_AE
CYM015	M	32	628	961	5.2	R5	CRF 01_AE
BJMSM376	M	54	598	1,198	5.9	R5	CRF 01_AE
020100259	M	32	506	1,292	5.7	R5	B′
020100104	F	52	421	943	3.9	X4/R5	B′
020100968	M	38	440	1,093	5.2	R5	B′
020100096	F	44	141	468	4.4	R5	B′
020100311	M	39	552	1,159	5.0	X4/R5	B′

### Phenotypic susceptibility of different HIV-1 subtypes to HIV entry inhibitors

TZM-bl cells were infected with different subtypes of HIV-1 isolates in the presence of various concentrations of the HIV entry inhibitors (enfuvirtide, C34, TAK779, maraviroc and AMD3100). Luciferase activity was measured for evaluation of the viral infectivity and IC_50_ value for each inhibitor was calculated. All the isolates were susceptible to HIV fusion inhibitors and CCR5 antagonists with average IC_50_ values of 60±66, 19±12, 78±59 and 1.40±1.36 nM for enfuvirtide, C34, TAK779 and maraviroc, respectively. The three subtypes of HIV-1 isolates possessed similar susceptibility to C34, while they exhibited different sensitivity to enfuvirtide, TAK779 and maraviroc. CRF07_BC isolates were about 5-fold less sensitive than CRF01_AE, B′ and B isolates to enfuvirtide, whereas the subtype B′ and B isolates were 4- to 5-fold more sensitive than CRF07_BC and CRF01_AE to TAK779 and maraviroc. It is noticeable that there was a high variation in the data obtained from testing different strains in the same subtype, which may result from the presence of random error in viral titration and susceptibility testing, the gene diversity and polymorphism in the same subtype, and the relatively small sample size. However, the difference of susceptibilities to enfuvirtide between CRF07_BC and CRF01_AE or B′ isolates and that to TAK779 and maraviroc between subtype B′ and CRF07_BC or CRF01_AE were statistically significant (*P*<0.05 or 0.01) when the Wilcoxon rank sum test was used. The CXCR4 antagonist AMD3100 at the concentration as high as 5 µM exhibited no significant inhibitory activity against any of the isolates tested, including 2 dual-tropic (X4/R5) strains (Table 2).

**Table 2:** Baseline susceptibility of HIV-1 clinical isolates that are predominantly circulating in China to HIV entry inhibitors.

Virus	HIV-1 entry inhibitors targeting				
	NHR of gp41		CCR5		CXCR4
	enfuvirtide	C34	TAK779	maraviroc	AMD3100
Subtype CRF 07_BC					
CBJB257	0.076±0.017	0.009±0.002	0.199±0.160	1.444±0.157	>5
XJDC6291	0.114±0.077	0.019±0.001	0.112±0.023	1.729±0.103	>5
CBJB256	0.054±0.014	0.016±0.005	0.061±0.047	1.140±0.120	>5
XJDC6371	0.169±0.029	0.014±0.001	0.078±0.036	4.948±1.495	>5
XJDC6331	0.226±0.030	0.042±0.013	0.092±0.023	2.254±0.127	>5
Mean	0.128±0.033	0.020±0.004	0.108±0.058	2.303±0.400	>5
Subtype CRF 01_AE					
CYM033	0.023±0.007	0.026±0.005	0.162±0.049	2.978±1.141	>5
BJMSM543	0.052±0.027	0.017±0.005	0.041±0.008	0.515±0.137	>5
CYM015	0.013±0.001	0.005±0.000	0.151±0.030	0.261±0.033	>5
BJMSM376	0.011±0.005	0.013±0.003	0.075±0.013	2.369±0.38	>5
Mean	0.025±0.010	0.015±0.003	0.107±0.025	1.531±0.423	>5
Subtype B′					
020100259	0.034±0.019	0.052±0.017	0.014±0.002	0.510±0.000	>5
020100104	0.013±0.003	0.012±0.003	0.040±0.003	0.204±0.021	>5
020100968	0.036±0.003	0.013±0.005	0.012±0.004	0.399±0.121	>5
020100096	0.012±0.001	0.016±0.000	0.016±0.000	0.260±0.000	>5
020100311	0.011±0.005	0.013±0.003	0.034±0.007	0.584±0.054	>5
Mean	0.021±0.006	0.021±0.007	0.023±0.003	0.391±0.039	>5

Each sample was tested in triplicate, and each experiment was repeated twice. IC_50_ (µM, except nM for maraviroc) data are presented as means ± standard deviations.

### The polymorphism analysis of the HIV-1 Env gp41 and gp120

**Figure 2:**
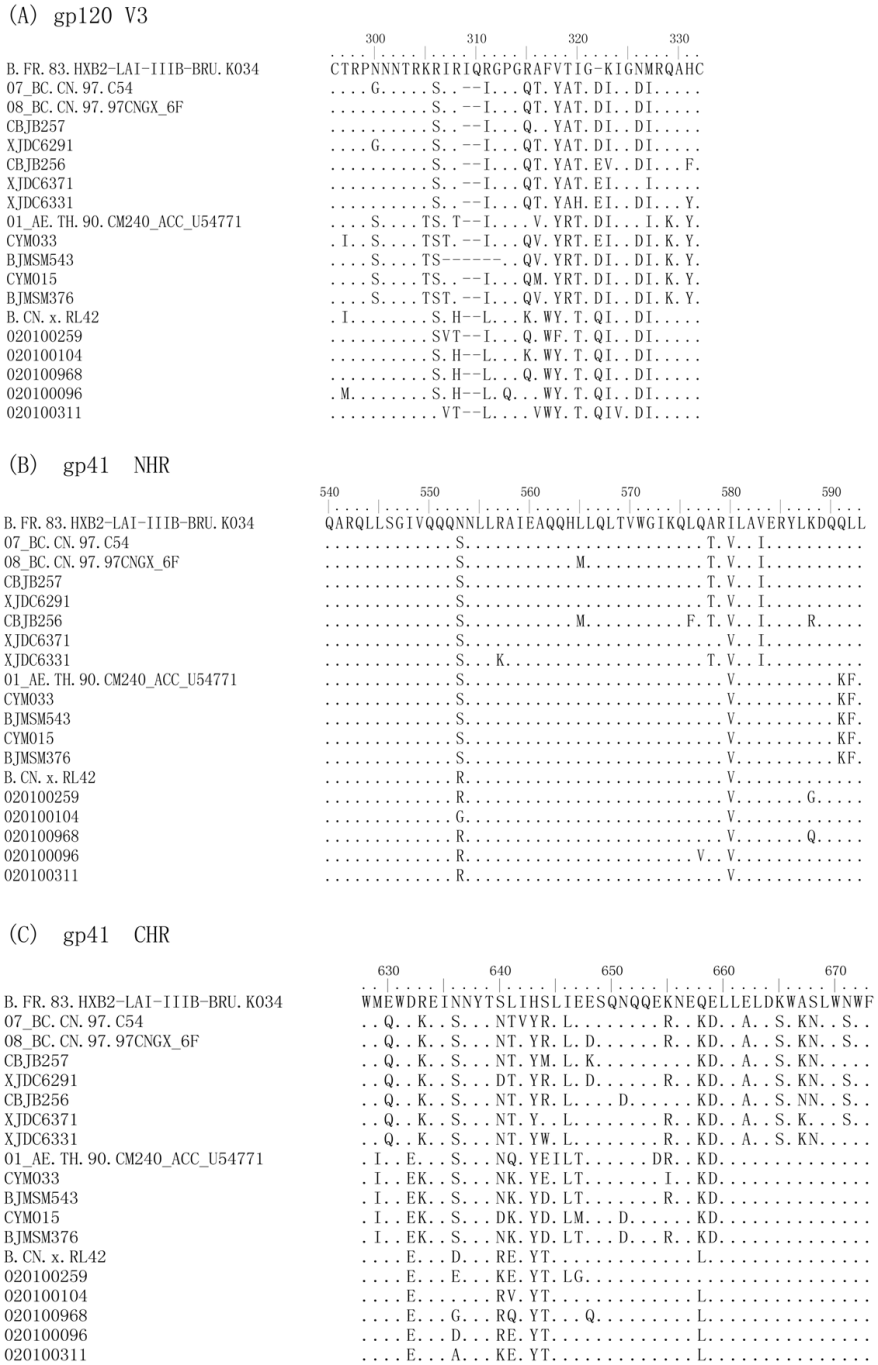
Alignment of the HIV-1 gp160. The V3 region and CHR sequences of the different CRFs and subtype B′ were highly polymorphic, compared to the B′ subtype. The NHR sequences were relatively conserved. HXB2-LAI-IIIB-BRU (Acc K03455), CN.97, C54, CN.97.97CNGX_6F, CM240_ACC_U54772 and RL42 were used as reference sequences.

The amino acid sequences of the viral Env gp41 and gp120 were analyzed and compared with the corresponding reference sequences in order to identify the substitutions and polymorphism possibly related with the natural resistance to the HIV entry inhibitors (Fig. 2). There was no visible substitution in the GIV tripeptide sequence (residues 547–549) of gp41 N-terminal heptad repeat (NHR), the primary determinant of viral resistance to enfuvirtide [18]. The polymorphism at position 553 was N553S in all the CRFs or N553R in most B′ isolates. The polymorphism at position 580 was I580V in all the isolates. Interestingly, the polymorphisms of A578T and V583I were only found in CRF07_BC strains, which were about 5-fold less sensitive to enfuvirtide than CRF01_AE and subtype B′ strains (Table 2). The polymorphisms of Q591K and L592F were found in all the CRF01_AE isolates.

The C-terminal heptad repeat (CHR) sequences of different CRFs were highly polymorphic, compared with the B′ subtype. In the region of residues 638–673 of CHR, which corresponds to the sequence of enfuvirtide, substitutions at positions 640, 641, 643, 644 and 658 were commonly recorded across the three different subtypes. The polymorphisms of E630Q, E662A, K665S, A667K and S668N in CHR of gp41 were found only in the CRF07_BC isolates.

High polymorphism was seen in the V3 region of gp120 from those B′, BC and AE isolates (Fig. 2). Polymorphisms of R306S, R311I, R315Q, V318Y, T319A, I320T, K323I, N326D, and M317I appeared in most of the CRF07_BC isolates, while the substitutions of N300S and K305T could only be seen in all the CRF01_AE isolates. Notably, the unique polymorphism of F317W was found in all subtype B′ isolates, which were about 4- to 5-fold more sensitive to CCR5 inhibitors, maraviroc and TAK779. Therefore, the two dominant polymorphism of R315Q and F317W in the V3 region of gp120 in CRF07_BC and CRF01_AE isolates might be related to their lower susceptibility to maraviroc and TAK779.

## Discussion

Since 2003, free ARVs, consisting of four NRTIs, one NNRTI, and one protease inhibitor, have been provided to rural residents and the urban poor with HIV infection in China [19], [20]. It has been shown that combinational use of these ARVs indeed results in significant reduction of mortality of the HIV/AIDS patients in China [13], [14]. However, increasingly more HIV/AIDS patients have failed to respond to these ARVs because of the emergence of drug-resistant HIV-1 variants [21], [22]. Therefore, it is essential to add some new classes of anti-HIV drugs into the ARV regimens used in China.

In recent years, two HIV entry inhibitors, enfuvirtide that targets the HIV-1 gp41 [11], [12] and maraviroc, a CXCR5 antagonist [23], [24] were approved by the US FDA for clinical use, but neither of them has been tested in China. Therefore, it is reasonable to question whether these HIV entry inhibitors are effective against the HIV-1 subtypes that are predominantly circulating in China. In this study, we intended to investigate the susceptibility of the HIV-1 subtypes that are predominantly circulating in China, including subtypes B′, CRF07_BC, and CRF01_AE, to the two newly marketed HIV entry inhibitors, enfuvirtide and maraviroc, and some other antiviral agents targeting HIV entry steps.

Here we found that these three subtypes of HIV-1 isolates possessed similar susceptibility to C34, but exhibited different sensitivity to enfuvirtide. CRF07_BC isolates were about 4- to 5-fold less sensitive to enfuvirtide than subtype B′ isolates (*P* = 0.004) and CRF01_AE isolates (*P* = 0.01). The NHR region in the gp41 ectodomain is the main target of enfuvirtide, C34, sifuvirtide (a C34 analog peptide under Phase II clinical trials), and other CHR peptides [25]–[28]. In the early *in vitro* study, enfuvirtide-induced viral resistance was associated with mutations in the GIV motif (residues 547–549) [18]. In the subsequent *in vivo* and *in vitro* studies, the determinant of resistance to enfuvirtide, sifuvirtide and C34 was mapped to the NHR domain of residues 547–556, which are critical for NHR and CHR interaction to form six-helix bundle core structure [29]–[32]. Some mutations in CHR (e.g., N637K, N648K, and S649A) may compensate for the loss in fitness and restore viral fusion kinetics while retaining the drug resistance[29]–[33].

To investigate why CRF07_BC isolates were less sensitive than subtype B′ and CRF01_AE isolates to enfuvirtide, we analyzed the gp41 NHR sequences. But we did not find any substitutions in the GIV motif and the residues 547–556 region in NHR related to viral resistance to enfuvirtide [18], [29]–[32], which is contrary to the report by Leung et al [34] who identified the G36D mutation in 19.4% of HARRT-experienced patients and 20.5% of ART-naïve patients in Hong Kong. The only polymorphism in this region is N553S, which was shown to be present in about 15% of HIV-1 isolates with non-decreased enfuvirtide susceptibility [35]. Interestingly, we found two unique polymorphisms beyond the region of residues 547–556 in NHR, A578T and V583I, which are unique to the CRF07_BC isolates (Fig. 2B). Further study is needed to investigate whether the polymorphisms A578T and V583I are associated with the natural viral resistance to enfuvirtide.

Different from the conserved NHR sequence, CHR of gp41 is highly polymorphic, particularly in the region corresponding to the T-20 synthetic peptide (residues 638–673). In this region, CRF07_BC is more polymorphic than CRF01_AE and B′, including A578T, V580I, E630Q, L641T, E662A, K665S, A667K and S668N. Whether these naturally occurring polymorphisms in the gp41 CHR of CRF07_BC strains are associated with their natural resistance to HIV entry inhibitors is unknown. Eggink et al [29] has proposed four mechanisms of viral resistance to enfuvirtide, including reduced contact, steric obstruction, electrostatic repulsion, and electrostatic attraction. For example, replacement of the non-charged residue in CHR with a charged residue, or vice visa, may cause electrostatic repulsion or attraction, resulting non-optimal helix packing and/or docking. In this study, we found several such substitutions, including E662A, K665S, and A667K, which are only present in the CRF07_BC. But it is unclear whether or not these substitutions are associated with the resistance of CRF07_BC to enfuvirtide.

We found that subtype B′ isolates were about 4- to 5-fold more sensitive than CRF07_BC isolates to TAK779 (*P* = 0.009) and maraviroc (*P* = 0.024), respectively. The two dual tropic viruses (020100104 and 020100311) of subtype B′ were also very sensitive to TAK779 and maraviroc. Recent studies have demonstrated that some of the dual tropic viruses mainly used CXCR4 for entry (dual-X4), while others are much more efficient in using the CCR5 (dual-R5) [36], [37]. Our preliminary result indicated that these two dual tropic viruses mainly used CCR5 co-receptor to enter cell, suggesting that they are dual-R5 strains. Polymorphic substitutions R311L/I, R315Q, A316T/V/M, T319A/R were found in V3 region of all CRFs and subtype B′. Multiple studies have demonstrated that the natural variation in the V3 loop affects the sensitivity of the HIV entry inhibitors, especially the substitutions in position 318 and 319. In our study, two dominant amino acid changes in V3 loop on gp120, R315Q and T319A, in CRF07_BC isolates may be associated with lower susceptibility to maraviroc, which is in agreement with several recent studies [38]–[40]. Subtype B′ isolates were also more sensitive than CRF01_AE isolates to TAK779 (*P* = 0.013) and maraviroc (*P* = 0.036), which may be associated with substitutions N300S, K305T and Q328K in V3 loop in gp120 of all CRF01_AE isolates. However, we could not exclude the possible maraviroc-resistant substitutions outside the V3 loop since the regions of the HIV-1 Env responsible for resistance to CCR5 inhibitors have mapped to not only V3 loop [41], but also other regions of the gp120 [42], or even the regions in gp41 [43].

AMD3100 exhibited no significant inhibitory activity against all the isolates tested even at 5 µM, which is more than 10,000-fold to the sensitive X4 strain. Though a paper reported that AMD3100 could inhibit infection by dual-tropic (X4/R5) strains [44], we did not find its inhibitory activity on the two dual-tropic strains isolated in this study. In a phase I/II clinical trial, AMD3100 was shown to significantly reduce HIV RNA levels in patients who harbored pure X4-tropic virus but not in the remaining subjects who harbored either dual-tropic or R5-tropic viruses [45]. Huang and colleagues reported that AMD3100 was able to suppress X4-tropic variants in *vivo*. The suppression of CXCR4-using variants by AMD3100 is dependent on both the tropism composition of the virus population and the efficiency of CXCR4 usage of individual variants. We thus assumed that the dual-tropic viruses isolated in our study may be more efficient in using CCR5 than CXCR4 to infect target cells.

In summary, CRF07_BC isolates were much less sensitive than B′ and CRF01_AE isolates to HIV fusion inhibitor, enfuvirtide, while subtype B′ isolates were more susceptible than CRF07_BC and CRF01_AE isolates to CCR5 antagonists, maraviroc and TAK779. The baseline resistance to the HIV entry inhibitors may be associated with the naturally occurring polymorphisms in these subtypes. This study thus provides useful information for rational design of optimal therapeutic regimens to treat patients infected with different HIV-1 subtypes.

## Methods

### Study population

The HIV-1 CRF07_BC and B′ isolates were isolated from the blood of pre-selected HIV-1-infected patients, who participated in a multicenter AIDS Cohort Study in China during 2003–2005 [46]. HIV-1 CRF_01AE isolates were from local HIV-1-positive individuals in Beijing, who were recruited to the AIDS Cohort Study in 2006–2008 as subjects infected through sexual transmission. The plasma viral loads of blood samples collected from 14 selected antiretroviral treatment-naïve HIV-1 infected patients were measured with COBAS AMPLICORTM techniques and analyzer (Roche Diagnostics, Alameda, CA). CD4^+^ T lymphocyte counts in whole blood were quantitated by flow cytometry using reagents and equipment provided by Becton Dickinson Biosciences (San Jose, CA). This study was approved by the Institutional Research Ethics Community, China CDC (IRB00002276), and all subjects signed informed consent forms before blood collection.

### HIV entry inhibitors

AMD3100, TAK779 and maraviroc were obtained from the National Institutes of Health AIDS Research and Reference Reagent Program. C34 and T-20 were synthesized by a standard solid-phase Fmoc (N-(9-fluorenyl-) methoxycarbonyl) method in the MicroChemistry Laboratory of the New York Blood Center (New York, NY).

### Isolation of HIV-1 from PBMCs from HIV-1-infected patients

PBMCs from patient's whole blood were isolated by standard density gradient centrifugation using Ficoll-Paque PLUS density gradient medium (GE Healthcare Bio-Sciences Corp., Piscataway, NJ). The isolated PBMCs were cocultured with phytohemagglutinin-stimulated PBMCs from at least 2 HIV-1 seronegative blood donors in RPMI-1640 medium supplemented with 10% fetal calf serum, 100 U/mL penicillin, 100 mg/mL streptomycin, 2.9 mg/mL L-glutamine and 100 IU rhIL-2 (Roche Diagnostics) as previously described [46]. The p24 antigen levels of culture supernatants were measured once per week for 4 weeks using a commercial enzyme-linked immunosorbent assay (ELISA) kit according to the manufacturer's recommendations (Bio-Merieux, Marcy-l'Etoile, France). Virus culture supernatants with p24 >1 ng/mL were aliquoted and stored in liquid nitrogen until use [47].

### HIV-1 Env sequence analysis

Viral RNA was extracted from virus isolated from HIV-1 patients using RNA extraction kit (Qiagen, Hilden, Germany). The viral RNA was used to generate reverse strand cDNA by RT-PCR kit (Invitrogen, Carlsbad, CA). The sequences of the Env region were then amplified by a nested polymerase chain reaction (nest-PCR) as previously described [48]. The outer primers for clades B′, CRF07_BC, and CRF01_AE were HZBOB/HZBOE pair, HZBCOB/HZBCOE pair and HZAEOB/HZAEOE pair, respectively, which yield approximately 3100-base pair (bp) products. The inner primers for clades B′, BC, and AE were HZBIB/HZBIE pair, HZBCIB/HZBCIE pair and HZAEIB/HZAEIE pair, respectively, which yield 2970-bp products [48]. The amplified fragments were identified on an agarose gel by electrophoresis, purified with QIAquick Gel Extraction kit (QIAGEN), directly sequenced using an ABI 377 Sequencer (Applied Biosciences) and then analyzed using Vector NCI software.

### Susceptibility assay to HIV entry inhibitors based on TZM-bl cells

The antiviral activity of HIV entry inhibitors against HIV-1 isolates from treatment-naïve patients was determined using TZM-b1 cells as previously described [49]. The 50% tissue culture infectious dose (TCID_50_) of a single thawed aliquot of each virus was determined in TZM-bl cells as described previously [50]. TZM-bl cells were seeded (10^4^/well) and cultured in a 96-well tissue culture plate overnight. The following day, 100 TCID_50_ diluted virus was added per well in the absence or presence of drugs with five series dilutions (5, 1, 0.1, 0.01 and 0.001 µM for enfuvirtide, C34, TAK779 and AMD3100; 1, 0.1, 0.01, 0.001 and 0.0001 µM for maraviroc). After further incubation at 37°C for 48 hours, the luciferase activity was measured using luciferase assay regents (Promega) and a Luminescence Counter (Perkin-Elmer) according to the manufacturer's instructions. The concentration of drug that inhibits 50% viral replication (IC_50_) was determined by nonlinear regression using GraphPad Prism 5.01. Mean IC_50_s were calculated using all replicates for each virus and are expressed as mean± SD.

### Statistical Methods and Analysis

The Wilcoxon rank sum test was applied to pairwise comparisons to determine whether the observed differences between IC_50_ for different subtype were statistically significant. The value of 0.05 was used as the criterion for statistical significance.
